# Diabetes mellitus increases risk of adverse drug reactions and death in hospitalised older people: the SENATOR trial

**DOI:** 10.1007/s41999-023-00903-w

**Published:** 2023-12-21

**Authors:** Anagha Chinmayee, Selvarani Subbarayan, Phyo Kyaw Myint, Antonio Cherubini, Alfonso J. Cruz-Jentoft, Mirko Petrovic, Adalsteinn Gudmundsson, Stephen Byrne, Denis O’Mahony, Roy L. Soiza

**Affiliations:** 1 Ageing Clinical and Experimental Research (ACER) Group, Institute of Applied Health Sciences, University of Aberdeen, Aberdeen, UK; 2 Aberdeen Royal Infirmary, NHS Grampian, Aberdeen, Scotland, UK; 3 Geriatria, Accettazione Geriatrica e Centro Di Ricerca Per L’invecchiamento, IRCCS INRCA, Ancona, Italy; 4 Servicio de Geriatría, Hospital Universitario Ramón y Cajal (IRICYS), Madrid, Spain; 5 Department of Geriatrics, Ghent University Hospital, Ghent, Belgium; 6 Landspitali University Hospital, Reykjavik, Iceland; 7 School of Pharmacy, University College Cork, Cork, Ireland; 8 Department of Medicine (Geriatrics), School of Medicine, University College Cork, Cork, Ireland

**Keywords:** Adverse drug reactions, Diabetes mellitus, Older people, Multimorbidity, Polypharmacy

## Abstract

**Purpose:**

Adverse drug reactions (ADRs) are a major cause of morbidity and mortality, especially in older people. Older people with diabetes mellitus may be at especially high risk of ADRs but this risk has not been well studied. This study aimed to compare severity and type of ADRs in hospitalised, multimorbid older people with and without diabetes and secondly to assess the impact of ADRs on mortality, rehospitalisation and length of stay.

**Methods:**

Participants in the SENATOR (Software Engine for the Assessment and optimization of drug and non-drug Therapy in Older peRsons) trial were assessed for 12 common and ‘other’ prevalent and incident adverse drug reactions using a blinded end-point adjudication process. Descriptive analyses, logistic regression and mediation analyses were undertaken.

**Results:**

Of 1537 people in the SENATOR trial, 540 (35.1%) had diabetes mellitus (mean age 77.4 ± 7.3 years, 58.5% male). In the total population, 773 prevalent and 828 incident ADRs were reported. Both prevalent and incident symptomatic hypoglycaemia and incident acute kidney injury (AKI) were significantly more common in people with diabetes (p < 0.05). Patients with diabetes had higher all-cause mortality at 12 weeks than those without (9.1% vs 6.3%, p = 0.04). Mediation analysis revealed that mortality was significantly higher (OR = 1.43, Sobel test p = 0.048) in people with diabetes and ADRs causing AKI.

**Conclusions:**

Older multimorbid people with diabetes presenting to hospital with acute illness have significantly more ADRs than those without, and a significantly higher mortality that is mediated by medication-associated AKI and poorer renal function.

**Supplementary Information:**

The online version contains supplementary material available at 10.1007/s41999-023-00903-w.

## Key summary points

**Aim:**

To compare severity and type of ADRs in hospitalised, multimorbid older people with and without diabetes and to assess the impact of ADRs on mortality, rehospitalisation and length of stay.

**Findings:**

Patients with diabetes had significantly more ADRs causing hypoglycaemia and acute kidney injury (AKI), with higher mortality that was mediated by drug-related AKI.

**Message:**

Clinicians should be especially aware of ADRs in people with diabetes, especially diuretics causing AKI.

**Supplementary Information:**

The online version contains supplementary material available at 10.1007/s41999-023-00903-w.

## Background

Adverse drug reactions (ADRs) are defined as “an appreciably harmful or unpleasant reaction, resulting from an intervention related to the use of a medicinal product, which predicts hazard from future administration and warrants prevention or specific treatment, or alteration of the dose regimen or withdrawal of the product” [1]. The most common risk factors for ADRs are age, multimorbidity and polypharmacy [2, 3]. Aside from being a major cause of morbidity and mortality, ADRs can have a significant impact on an individual’s quality of life and place an increased burden on healthcare systems due to higher patient care costs and prolonged hospital stays [4, 5]. Two systematic reviews reported a pooled ADR prevalence of 16% (26 studies including 20,153 patients) [6] and 22% (18 studies, involving 80,695 patients) [7] respectively in hospitalised older people and individual study prevalence ranged from 6.3 to 64.4%. A systematic review of 18 observational studies from European countries and the USA reported that costs due to preventable ADRs in an inpatient setting ranged from €2851 to €9015 per hospitalisation [8].

Diabetes mellitus is a chronic condition with a rising prevalence globally and is associated with greater mortality, decreased functional status, and increased hospitalisation among older people [9, 10]. According to 2021 data, it is estimated that 536.6 million people worldwide have diabetes, accounting for 10.5% of the global population with the highest prevalence of 19.3% seen in older people aged ≥ 65 years. According to recently reported data, nearly one in five older people has diabetes [11, 12]. Older people with diabetes often have multiple co-morbidities, and tend to be prescribed numerous medications [13–15]. As such, the risk of ADRs in this population is higher, not only due to anti-diabetic medications, but also due to the physiological changes that occur with old age and the micro- and macro-vascular complications of the condition [3, 7, 16] plus under-representation of older people from drug trials that would allow accurate estimation of the real risk of ADRs [17].

A cohort study using diary data conducted by Denig et al. in a primary care setting reported that out of 78 patients with diabetes mellitus almost half (46%) reported at least one ADR. Of the 80 ADRs reported, 71 (90%) were known ADRs based on the summary of product characteristics (SmPC) and no formal causality was assessed [18].

A few studies from low-and-middle-income countries have reported ADRs in the diabetic population in the hospital setting [19–23]. However, those studies were focused mainly on ADRs due to anti-diabetic medications or reported ADRs as part of drug-therapy problems (DTPs) in diabetes and were methodologically weak with most being observational studies. More importantly, these studies have not been carried out in the older population, despite diabetes being a condition that affects mostly older adults. To our knowledge, there is no study that has evaluated the risk of ADRs in hospitalised older people with diabetes.

The primary objective of this study was to compare the incidence, prevalence, severity, and type of ADR in hospitalised multimorbid older people with and without diabetes using data from the SENATOR trial. The secondary objectives were to assess the impact of ADRs in people with diabetes on mortality, rehospitalisation, and length of stay (LOS) using logistic regression and mediation analysis.

## Methods

### Study design

Analyses were carried out using data from the SENATOR (Software Engine for the Assessment and optimization of drug and non-drug Therapy in Older peRsons) trial, clinical trial registration number NCT02097654 (clinicaltrials.gov). The study was approved by each participating institution’s research ethics committee. The SENATOR protocol has been published previously [24]. In brief, SENATOR was a pragmatic, multi-national, parallel-arm prospective randomized open-label, blinded endpoint (PROBE) controlled trial that tested the impact of the SENATOR software tool in reducing in-hospital ADRs in multimorbid older people with polypharmacy. The study was conducted between July 2016 to February 2018 and included a diverse population from six academic teaching hospitals across Europe (Belgium, Iceland, Ireland, Italy, Spain and United Kingdom) [24, 25].

### Study population

The trial included older people aged ≥ 65 years with ≥ three chronic medical conditions requiring pharmacotherapy who were admitted to the hospital with acute medical or surgical illness. The main exclusion criteria were (i) elective admission, (ii) admission to geriatric medicine, clinical pharmacology, clinical oncology, haematology, psychiatry, palliative medicine, emergency medicine, intensive care units, (iii) acute liver failure, (iv) renal failure requiring dialysis, (v) solid organ transplant graft, (vi) non-accidental overdose/self-harm, (vii) estimated life expectancy less than three months; (viii) anticipated discharge/hospital transfer within 48 h of admission and (ix) admitted to hospital > 60 h at the time of planned enrolment [24, 25].

### Definition and adjudication of ADRs

SENATOR gathered data on both prevalent and incident ADRs in the trial. Prevalent ADRs were defined as the ADR events/processes that were either the primary cause or partly the cause of hospitalization or which occurred in the emergency department or other locations in the hospital up to the time of randomization. Incident ADRs were defined as ADR events/processes that occurred between randomization and the index hospital discharge or day 14 post-randomisation, whichever occurred first. The ADRs were reported using a study-specific endpoint form based on a review of all the available documentation within the medical record including medical, nursing and allied health professional case note entries, laboratory values, radiology reports, electrocardiograms and other investigations [24, 25].

The SENATOR trial defined 12 pre-specified ADRs that represent approximately 80% of all ADRs commonly reported in hospitalised multimorbid older people. The 12 pre-specified ADRs were acute bleeding, acute diarrhoea, new-onset constipation, acute dyspepsia/nausea/vomiting, acute kidney injury (AKI), symptomatic hypoglycaemia (SH), new-onset fall/s, delirium, major serum electrolyte disturbance, symptomatic bradycardia, symptomatic orthostatic hypotension and new-onset unsteady gait. Any non-pre-specified ADRs were documented as unspecified adverse events [24, 25]. Therefore, a total of 13 types of ADRs were reported in the SENATOR trial. The definitions of the pre-specified ADRs are provided in Supplementary Table 1.

The ADRs reported using the study-specific endpoint assessment form (ADR form) were adjudicated by a blinded Potential Endpoints Adjudication Committee which ascertained the causality and severity of the ADR. The committee consisted of six blinded expert members who reviewed ADRs independently. If the first blinded endpoint committee reviewer agreed with the site principal investigator’s record of causality and severity, the decision was accepted. If not, the ADR forms were reviewed by the second reviewer. If there was no agreement after the second review, the ADR forms were reviewed by the third reviewer and a consensus was reached. Otherwise, the ADR form was adjudicated by consensus at a full committee meeting excluding the site Principal Investigator [24, 25]. Causality of ADRs was assessed using the WHO-UMC causality assessment system which categorized ADRs as certain, probable, possible, unlikely and indeterminate [26] and ADR severity was graded as mild, moderate and severe according to the modified Hartwig & Siegel severity assessment scale [27].

### Data handling

The SENATOR trial included data from three assessments i.e., baseline, discharge or day 14 whichever occurred first and a 12-week follow-up visit. For this study purpose, patients were categorised based on the presence or absence of diabetes from their medical history (ICD-10 classification). We included patients with both Type 1 and Type 2 diabetes mellitus. The variables of interest included demographic variables (recruiting centre, age, sex, smoking and alcohol use and level of education); clinical variables (admitting ward, the total number of medications, number of co-morbidities, incidence, prevalence, severity and causality of ADRs and laboratory values such as eGFR and albumin; functional, comorbidity status and cognitive rating scale status (Barthel Index of activities of daily living, Cumulative Illness Rating Scale-Geriatric (CIRS-G) and Mini-Mental State Examination score (MMSE)); and secondary outcome variables (mortality, rehospitalisation and length of stay (LOS)).

### Compilation of ADRs data

The primary outcome variables included severity and causality of prevalent and incident ADRs. From the total list of unadjudicated ADRs collected in the trial, the SENATOR team excluded those suspected ADRs where no culprit drug was identified and the ADRs with no data for severity and causality before adjudication. After adjudication, the ADRs were deemed non-eligible if the severity rating was missing or graded zero and only eligible adjudicated ADRs were included in our analyses. Although the SENATOR trial data had five categories for causality, to ensure an adequate study power for our analysis, we categorised ADRs into two groups for causality i.e., indeterminate/unlikely/possible and probable/certain. This was done because the numbers of indeterminate and unlikely events were small. Similarly, the severity was grouped into two categories, mild and moderate/severe.

### Potential confounders and mediators

Using standard criteria to identify confounders [28], age, sex, Barthel Index score, CIRS-G score, smoking, alcohol, number of medications and number of co-morbidities were included as confounders. Of the 12 pre-specified ADRs, only those that were clinically relevant to diabetes and those that showed significance between people with and without diabetes (AKI and SH) were considered confounders in the logistic regression analysis.

A mediator is a variable that is in a causal sequence between two variables, the independent and dependent variables [29]. We considered the presence/absence of diabetes as our independent variable and mortality at 12 weeks, rehospitalisation at 12 weeks of discharge and LOS as our dependent variables. Based on this definition, the variables chosen as mediators were eGFR, serum albumin concentration and the 12 pre-specified ADRs.

### Statistical analysis

IBM SPSS Statistics version 27.0 was used for data analysis. Depending on the data distribution, continuous data were presented as median (interquartile range, IQR) or mean ± standard deviation (SD). In addition to visual inspection of the distribution, skewness of minus one to plus one was considered normal distribution. Categorical data were presented as frequencies and proportions. Group differences between people with and without diabetes for continuous variables were analysed using parametric (unpaired t-test) and nonparametric (Mann–Whitney *U* test) tests respectively for normal and skewed data distribution. Categorical data were compared using Chi-square or Fisher’s exact tests.

Logistic regression analysis was used to examine the relationship between having diabetes and the impact of ADRs on outcomes i.e., mortality, rehospitalisation and LOS. Models were built with a stepwise approach using multivariable logistic regression, adjusting for confounders one at a time and adding ADRs (AKI and SH) in the final model. LOS was dichotomised as ≤ / > 6 as 6 days was the median LOS. Odds ratios with 95% confidence intervals and p-values were reported.

Mediation analysis was performed according to the framework proposed by Baron and Kenny [30] to assess whether mediators explain differences in outcomes in people with and without diabetes. The criteria for analysis included: Step (*1)* the independent variable must be significantly related to the dependent variable; Step (*2*) the independent variable must be significantly related to the mediator, and Step (*3*) the association between the independent and dependent variable must be attenuated when the mediator is included in the regression model [28]. The indirect effect was calculated as a*b and c is the direct effect. Sobel’s test was used to determine the significance of the effect. For all analyses, a two-sided p-value of < 0.05 was considered statistically significant.

## Results

### Participant characteristics

A total of 1537 participants were recruited to the SENATOR trial; 405 (26.4%) from Cork, 295 (19.2%) from Reykjavik, 285 (18.5%) from Aberdeen, 205 (13.3%) from Ghent, 190 (12.4%) from Madrid and 157 (10.2%) from Ancona. The mean age of the total population was 78.2 ± 7.4 years with 52.8% being male.

Of 1537 people included in our analysis, 540 (35.1%) participants had diabetes. The mean age of people with diabetes was 77.4 ± 7.3 years and 78.6 ± 7.5 years for people without diabetes (p = 0.002). In the group with diabetes, 316 (58.5%) were male compared to 496 males (49.7%) in the group without diabetes (p = 0.001). The median number of medications was significantly greater in people with diabetes (10 [8, 12] versus 9 [6, 11]; p < 0.001), as was the number of co-morbidities (12 [8, 16] versus 9 [7, 13], p < 0.001). In addition, both the CIRS-G score (16.5 ± 5.8 vs. 14.4 ± 5.7, p < 0.001) and Barthel index score (18 (14,20) vs. 18 (14,20), p = 0.004) were significantly different between the two groups, with the diabetes group having greater burden of morbidity and disability. Participant characteristics are presented in Table 1.

**Table 1:** Participant characteristics of people with and without diabetes

Variable	Total population (n = 1537) n (%)	People with diabetes (n = 540) n (%)	People without diabetes (n = 997) n (%)	p-value
Recruiting centre				< 0.001
Aberdeen	285 (18.5%)	120 (42.1%)	165 (57.9%)	
Ancona	157 (10.2%)	56 (35.7%)	101 (64.3%)	
Cork	405 (26.4%)	104 (25.7%)	301(74.3%)	
Ghent	205 (13.3%)	85 (41.2%)	120 (58.8%)	
Madrid	190 (12.4%)	85 (44.7%)	105 (45.3%)	
Reykjavik	295 (19.2%)	90 (30.5%)	205 (69.5%)	
Age years (mean ± SD)	78.2 ± 7.4	77.4 ± 7.3	78.6 ± 7.5	0.002
Sex				0.001
Male	812 (52.8%)	316 (58.5%)	496 (49.7%)	
Female	725 (47.2%)	224 (41.5%)	501(50.3%)	
Smoking status				0.41
Yes	108 (7%)	34 (6.3%)	74 (7.4%)	
No	1,429 (93%)	506 (93.7%)	923 (92.6%)	
Alcohol				0.06
Yes	432 (28.1%)	136 (25.2%)	296 (29.7%)	
No	1,105 (71.9%)	404 (74.8%)	701 (70.3%)	
Education				0.74
No schooling	37 (2.4%)	12 (2.2%)	25 (2.5%)	
Primary school education only	561 (36.5%)	198 (36.7%)	363 (36.4%)	
Some secondary education	281 (18.3%)	89 (16.5%)	192 (19.3%)	
Complete secondary education	448 (29.1%)	165 (30.6%)	283 (28.4%)	
Some third-level education	55 (3.6%)	22 (4.1%)	33 (3.3%)	
Complete third-level education	155 (10.1%)	54 (10%)	101 (10.1%)	
Previous documented ADR(s)	669 (43.5%)	224 (41.5%)	445 (44.6%)	0.23
Total number of medications median [IQR]	9 [7, 11]	10 [8, 12]	9 [6, 11]	< 0.001
Number of medical conditions median [IQR]	10 [7, 14]	12 [8, 16]	9 [7, 13]	< 0.001
Admitted ward				0.20
Medical	1299 (84.5%)	465 (86.1%)	834 (83.7%)	
Surgical	238 (15.5%)	75 (13.9%)	163 (16.3%)	
Patients who experienced a prevalent ADR				0.56
Yes	563 (36.6%)	203 (37.6%)	360 (36.1%)	
No	974 (63.4%)	337 (62.4%)	637 (63.9%)	
Patients who experienced an incident ADR				0.17
Yes	551 (35.8%)	206 (38.1%)	345 (34.6%)	
No	986 (64.2%)	334 (61.9%)	652 (65.4%)	
CIRS-G score (mean ± SD)	15.1 ± 5.9	16.5 ± 5.8	14.4 ± 5.7	< 0.001
Barthel Index ADL (median [IQR])	18 [14, 20]	18 [14, 20]	18 [14, 20]	0.004
MMSE score (median [IQR])*	27 [23, 29]	27 [23, 29]	27 [24, 29]	0.70

*SD* standard deviation, *IQR* interquartile range, *ADRs* adverse drug reactions, *MMSE* mini-mental state examination, *CIRS-G* cumulative illness rating scale-geriatric (CIRS-G) score, *Barthel Index ADL* barthel index of activities of daily living

*Total population included in the analysis was 1503 for MMSE score

### Primary outcomes

#### Prevalence and incidence of ADRs in the SENATOR data

A total of 3247 unadjudicated putative ADRs were reported in the SENATOR trial; 886 putative ADRs were excluded as no culprit drugs were identified. Of the remaining 2361 putative ADRs, there were 1080 (45.7%) prevalent and 1281 (54.3%) incident ADRs. Of 1080 unadjudicated prevalent ADRs, 17 ADRs with missing data for causality and severity were removed and 1063 were sent for adjudication. After adjudication, 290 were determined as being non-eligible (i.e. not an ADR) leaving 773 eligible prevalent ADRs (see Supplementary Fig. 1). Of the 1281 unadjudicated incident ADRs, 232 ADRs with missing data for causality and severity were excluded prior to adjudication and 1049 were sent to the adjudication committee. Following adjudication, 221 were considered non-eligible leaving 828 eligible incident ADRs (see Supplementary Fig. 2).

### Comparison of prevalent ADRs between people with and without diabetes

Of 773 prevalent ADRs, 284 (36.7%) were observed in people with diabetes versus 489 (63.3%) ADRs in people without diabetes (see Table 2). Of 1537 participants, 203 (37.6%) participants with diabetes versus 360 (36.1%) participants without diabetes experienced a prevalent ADR, p = 0.56 (see Table 1).

**Table 2:** Comparison of severity of prevalent ADRs between people with and without diabetes

Prevalent ADRs	Total no. of prevalent ADRs (n = 773) n (%)	Prevalent ADRs in people with diabetes (n = 284) n (%)		Prevalent ADRs in people without diabetes (n = 489) n (%)		p-value
		Mild	Moderate/severe	Mild	Moderate/severe	
Acute bleeding	80 (10.3)	4 (1.4)	16 (5.6)	27 (5.5)	33 (6.7)	0.05
Acute diarrhoea	30 (3.9)	6 (2.1)	8 (2.8)	9 (1.8)	7 (1.4)	0.18
New onset constipation	42 (5.4)	2 (0.7)	9 (3.2)	5 (1)	26 (5.3)	0.22
Acute dyspepsia/nausea/vomiting	59 (7.6)	4 (1.4)	13 (4.6)	13 (2.7)	29 (5.9)	0.30
Acute kidney injury (AKI)	82 (10.6)	20 (7)	13 (4.6)	26 (5.3)	23 (4.7)	0.32
Symptomatic hypoglycaemia (SH)	14 (1.8)	1 (0.4)	12 (4.2)	0	1 (0.2)	< 0.001
New onset fall/S	110 (14.2)	13 (4.6)	23 (8.1)	17 (3.5)	57 (11.7)	0.58
Delirium	39 (5.0)	4 (1.4)	10 (3.5)	10 (2)	15 (3.1)	0.92
Major serum electrolyte disturbance	176 (22.8)	32 (11.3)	37 (13)	34 (7)	73 (14.9)	0.23
Symptomatic bradycardia	25 (3.2)	1 (0.4)	5 (1.8)	7 (1.4)	12 (2.5)	0.24
Symptomatic orthostatic hypotension	17 (2.2)	3 (1.1)	4 (1.4)	4 (0.8)	6 (1.2)	0.60
New onset unsteady gait	19 (2.5)	4 (1.4)	5 (1.8)	5 (1)	5 (1)	0.26
Unspecified adverse event	80 (10.3)	15 (5.3)	20 (7)	19 (3.9)	26 (5.3)	0.10

*ADRs* adverse drug reactions

Among 12 different pre-specified ADRs, SH was the only prevalent ADR with a statistically significant difference between the groups, with 13 events in people with diabetes compared to 1 SH event in people without diabetes (p < 0.001). Insulin preparations were implicated in most cases, with glimepiride, gliclazide, sitagliptin and metformin contributing in five cases. Of the 13 SH, one SH was mild and 12 were moderate/severe compared to only one moderate/severe SH in people without diabetes, attributed as a possible ADR to trimethoprim and sulphamethoxazole. Instances of mild AKI, falls and electrolyte disturbance, and moderate/severe delirium were more common in people with diabetes, but these differences did not reach statistical significance. Interestingly, the percentage of both mild and moderate/severe unspecified ADRs was higher in people with versus people without diabetes, (p = 0.006).

In terms of causality, only SH showed a statistically significant difference between the groups, 12 were probable/certain, and one was indeterminate/unlikely/possible in people with diabetes compared to just one indeterminate/unlikely/possible event in people without diabetes. People with diabetes had more indeterminate/unlikely/possible AKI and electrolyte disturbance as well as a higher proportion of probable/certain electrolyte disturbance, though none of these differences reached statistical significance. The severity and causality of prevalent ADRs are presented in Table 2 and Supplementary Table 2 respectively.

#### Comparison of incident ADRs between people with and without diabetes

Of 828 incident ADRs, 334 (40.3%) were observed in people with diabetes and 494 (59.7%) in those without diabetes (see Table 3). Of 1537 participants, 206 (38.1%) participants with diabetes suffered incident ADRs compared to 345 (34.6%) participants without diabetes (p = 0.17) (see Table 1).

**Table 3:** Comparison of severity of incident ADRs between people with and without diabetes

Incident ADRs	Total no. of incident ADRs (n = 828) n (%)	Incident ADRs in people with diabetes (n = 334) n (%)		Incident ADRs in people without diabetes (n = 494) n (%)		p-value
		Mild	Moderate/severe	Mild	Moderate/severe	
Acute bleeding	68 (8.2)	17 (5.1)	9 (2.7)	23 (4.7)	19 (3.8)	0.58
Acute diarrhoea	65 (7.9)	22 (6.6)	5 (1.5)	28 (5.7)	10 (2)	0.27
New onset constipation	138 (16.7)	7 (2.1)	34 (10.2)	18 (3.6)	79 (16)	0.16
Acute dyspepsia/nausea/vomiting	68 (8.2)	6 (1.8)	21 (6.3)	17 (3.4)	24 (4.9)	0.42
Acute kidney injury (AKI)	99 (11.9)	20 (6)	27 (8.1)	24 (4.9)	28 (5.7)	0.008
Symptomatic hypoglycaemia (SH)	14 (1.7)	2 (0.6)	12 (3.6)	0	0	< 0.001
New onset fall/S	22 (2.7)	6 (1.8)	4 (1.2)	9 (1.8)	3 (0.6)	0.31
Delirium	44 (5.3)	11 (3.3)	8 (2.4)	17 (3.4)	8 (1.6)	0.26
Major serum electrolyte disturbance	163 (19.7)	24 (7.2)	31 (9.3)	43 (8.7)	65 (13.2)	0.69
Symptomatic bradycardia	11 (1.3)	1 (0.3)	1 (0.3)	4 (0.8)	5 (1)	0.24
Symptomatic orthostatic hypotension	16 (1.9)	2 (0.6)	4 (1.2)	8 (1.6)	2 (0.4)	0.84
New onset unsteady gait	3 (0.4)	1 (0.3)	0	1 (0.2)	1 (0.2)	1.00
Unspecified adverse event	117 (14.1)	28 (8.4)	31 (9.3)	24 (4.9)	34 (6.9)	0.006

*ADRs* adverse drug reactions

There were 20 (6%) mild AKI and 27 (8.1%) moderate/severe AKI that occurred in patients with diabetes compared to 24 (4.9%) mild and 28 (5.7%) moderate/severe AKI in patients without diabetes (p = 0.008). The culprit medications are listed in Table 4. Additionally, two mild and 12 moderate/severe SH were identified in people with diabetes compared to zero SH in those without diabetes (p < 0.001). Mild bleeding and mild diarrhoea were more common in people with diabetes. Similarly, people with diabetes had a greater percentage of moderate/severe dyspepsia/nausea/vomiting, falls, delirium and orthostatic hypotension although the difference was not statistically significant.

**Table 4:** Number of instances of certain and probable acute kidney injury by medication

Causative medications	Acute kidney injury			
	Certain and probable adverse drug reactions			
	Prevalent		Incident	
	People with diabetes (n = 33)	People without diabetes (n = 49)	People with diabetes (n = 47)	People without diabetes (n = 52)
Furosemide	6	11	20	21
Spironolactone	2	0	7	4
Bumetanide	1	2	4	1
Metolazone	1	0	2	3
Flucloxacillin	1	0	–	–
Gentamicin	0	1	0	2
Co-trimoxazole	0	1	1	1
Ramipril	0	1	3	0
Potassium canrenoate	1	2	1	3
Perindopril	1	0	–	–
Piroxicam	0	1	–	–
Dexketoprofen	0	1	–	–
Torsemide	0	1	–	–
Enalapril	2	0	0	2
Etoricoxib	0	1	–	–
Vancomycin	0	1	–	–
Radiology contrast	–	–	3	1
Metformin	–	–	1	0
Clindamycin	–	–	0	1
Piperacillin + Tazobactam	–	–	1	1
Co-amoxiclav	–	–	0	1
Canrenone	–	–	0	2
Mefenamic acid	–	–	0	1
Naproxen	–	–	1	1
Amlodipine	–	–	0	1
Hydrochlorothiazide	–	–	0	1

In terms of ADR causality, 20 AKI events were unlikely/indeterminate/possible and 27 AKI events were probable/certain in people with diabetes compared to 26 AKI events that were unlikely/indeterminate/possible and 26 AKI events that were probable/certain in people without diabetes. All 14 SH in people with diabetes were probable/certain (p < 0.001). In addition, a higher percentage of people with diabetes suffered indeterminate/unlikely/possible bleeding and constipation, and probable/certain diarrhoea, falls, and delirium in comparison to those without diabetes. Nevertheless, causality was not significantly different between the groups for any ADRs except SH. The severity and causality of incident ADRs are presented in Table 3 and Supplementary Table 3.

#### Impact of ADRs in diabetic patients on secondary outcomes

All-cause mortality at 12 weeks was higher in people with diabetes, 9.1% (46/505) compared to people without diabetes, 6.3% (59/944), p = 0.045. The number of people re-hospitalised at 12 weeks was 164 (36%) among those that had diabetes compared to 310 (35.4%) in people without diabetes (p = 0.84). LOS was the same in both the groups (6 [3, 11] vs 6 [3, 10], p = 0.36). The results for secondary outcomes are presented in Table 5.

**Table 5:** Secondary outcomes: comparison of mortality, rehospitalisation and length of stay between people with and without diabetes

Variable	Total population (n)	Total (n)	People with diabetes (n = 505)	People without diabetes (n = 944)	p-value
Mortality (all-cause) at 12 weeks	1449	105 (7.2%)	46 (9.1%)	59 (6.3%)	0.045
			People with diabetes (n = 456)	People without diabetes (n = 876)	
Re-hospitalisation (all-cause) at 12 weeks	1332	474 (35.6%)	164 (36%)	310 (35.4%)	0.84
			People with diabetes (n = 534)	People without diabetes (n = 984)	
Length of stay (median [IQR])	1518	6 [3, 11]	6 [3, 11]	6 [3, 10]	0.36

*IQR* interquartile range

The unadjusted model showed a statistically significant association between having diabetes and mortality (OR 1.50, 95% CI 1.01–2.25, p = 0.047). Multiple logistic regression showed a significant association remained when adjusted for age, sex and Barthel Index score (OR 1.55, 95% CI 1.02–2.34 and p = 0.039). The association did not reach statistical significance after adjustment for the burden of comorbidities. Furthermore, rehospitalisation and LOS did not show a significant association with having diabetes both in the unadjusted and multiple logistic regression models. The results for multiple logistic regression are shown in Table 6.

**Table 6:** Multiple logistic regression analysis showing the association between the presence of diabetes and mortality, rehospitalisation and length of stay

Model	Variables	Outcomes								
		Mortality			Rehospitalisation			Length of stay		
		OR	95% CI	p-value	OR	95% CI	p-value	OR	95% CI	p-value
unadjusted	Diabetes—Yes	1.50	1.01–2.25	0.047	0.98	0.77–1.24	0.84	0.99	0.80–1.22	0.90
A	Age	1.64	1.09–2.47	0.017	0.99	0.78–1.26	0.94	0.98	0.79–1.21	0.83
B	Age, sex	1.63	1.08–2.45	0.019	1.02	0.80–1.30	0.87	0.97	0.78–1.20	0.75
C	Age, sex, barthel index	1.55	1.02–2.34	0.039	1.05	0.83–1.33	0.70	1.02	0.82–1.26	0.87
D	Age, sex, barthel index, CIRSG—score	1.39	0.91–2.12	0.13	1.08	0.85–1.38	0.52	0.99	0.79–1.23	0.91
E	Age, sex, barthel index, CIRSG—score, smoking	1.38	0.90–2.10	0.14	1.08	0.85–1.38	0.54	0.99	0.79–1.23	0.91
F	Age, sex, barthel index, CIRSG—score, smoking, alcohol	1.36	0.89–2.07	0.16	1.08	0.84–1.38	0.56	0.98	0.79–1.22	0.86
G	Age, sex, barthel index, CIRSG—score, smoking, alcohol, no. of meds	1.38	0.90–2.12	0.14	1.14	0.89–1.46	0.30	1.01	0.81–1.27	0.91
H	Age, sex, barthel index, CIRSG – score, smoking, alcohol, no of meds, no. of co-morbidities	1.33	0.86–2.06	0.19	1.20	0.93–1.55	0.16	0.96	0.76–1.20	0.71
I	Age, sex, barthel index, CIRSG—score, smoking, alcohol, no. of meds, no. of co-morbidities, AKI (incident)	1.27	0.82–1.97	0.29	1.21	0.94–1.56	0.14	1.00	0.79–1.26	0.97
J	Age, sex, barthel index, CIRSG—score, smoking, alcohol, no of meds, no. of co-morbidities, AKI (prevalent)	1.32	0.85–2.04	0.22	1.20	0.93–1.55	0.16	0.96	0.76–1.20	0.71
K	Age, sex, barthel index, CIRSG—score, smoking, alcohol, no of meds, no. of co-morbidities, SH (incident)	1.34	0.86–2.07	0.19	1.19	0.92–1.54	0.18	0.99	0.79–1.24	0.91
L	Age, sex, barthel index, CIRSG—score, smoking, alcohol, no of meds, no. of co-morbidities, SH (prevalent)	1.35	0.87–2.09	0.18	1.21	0.94–1.56	0.15	0.96	0.76–1.20	0.70

*CIRS-G* cumulative illness rating scale-geriatric, *AKI* acute kidney injury, *SH* symptomatic hypoglycaemia, *OR* odds ratio, *CI* confidence interval

#### Mediation analysis

When testing the mediator role of ADRs in the relationship between having diabetes and mortality, in the first regression step, presence of diabetes was significantly associated with mortality (B = 0.41, p = 0.047). In the second step, presence of diabetes was positively associated with incident AKI (B = 0.55, p = 0.008). Finally, in the third step, when presence of diabetes and AKI were simultaneously included in the equation, having diabetes and incident AKI was significantly associated with mortality (OR = 1.43, Sobel test p = 0.048); the results are shown in Supplementary Fig. 3 and Supplementary Table 4. Similarly, eGFR fully mediated the association between presence of diabetes and mortality. Supplementary Fig. 4 and Supplementary Table 4 illustrate the findings. It was not possible to carry out mediation analysis for other outcomes i.e., rehospitalisation and LOS as the results were insignificant at Step 1. Furthermore, the other 12 ADRs and serum albumin concentration were not significant at Step 2 and hence could not be analysed further.

## Discussion

To the best of our knowledge, the present study is the first that specifically reports the burden of ADRs in an older hospitalized patient population with diabetes with specific details of both prevalent and incident ADRs in the hospital care setting. We found a higher rate of ADRs and an increased risk of all-cause mortality at 12 weeks in those with diabetes. The higher mortality rate was mediated by medication-associated AKI and lower eGFR. Diuretics were frequently implicated as the cause of medication-associated AKI.

Out of 1537 participants in the SENATOR trial, more than a third of people with diabetes experienced an ADR. An observational study that used data from 2257 hospitalized type 2 diabetes mellitus patients enrolled in the Gruppo Italiano di Farmacovigilanza nell’Anziano study, conducted in community and university hospitals across Italy from 1993 to 1998 reported that 10.2% of all patients had an ADR during the hospital stay. However, that study reported the incidence of ADRs due to hydrosoluble drugs in undiagnosed renal failure patients with diabetes and is now a considerably older study [31].

In the present study, although some particular ADRs occurred more frequently in people with diabetes, only AKI and SH reached statistical significance. Similarly, only SH showed significance amongst the prevalent ADRs, though this is not surprising. The unadjusted regression analysis showed a significant association between diabetes and mortality. Additionally, multiple logistic regression analysis showed this association remained significant when adjusted for age, sex and Barthel Index score. After adjustment for comorbidity burden, the association between mortality and diabetes remained but no longer reached statistical significance. This is probably because this association with death is partly driven by the accumulation of comorbidities, but most of these are themselves strongly associated with, or directly caused by, diabetes. Furthermore, mediation analysis confirmed that the mortality rates were significantly higher in patients with diabetes experiencing AKI (incident ADR) accounting for the difference in outcomes. However, diabetes does not appear to influence the likelihood of re-hospitalisation or duration of inpatient stay.

The higher risk of SH can very likely be attributed to a combination of tight glycaemic control, undernutrition and polypharmacy with drug-drug interactions with antidiabetic medication. Multiple studies [19, 32] identify hypoglycaemia as the most commonly observed ADR in people with diabetes but our study, with its highly detailed ADR ascertainment processes, shows that other ADRs are more prevalent.

People with diabetes are also at an increased risk of experiencing an AKI during hospitalization. This can be explained by the fact that diabetic patients incur variable degrees of kidney damage over time, exacerbated by nephrotoxic drugs. This is particularly significant in older people, as kidney function normally declines with age [31].

Our findings show how important it is to understand the burden of potentially avoidable ADRs and iatrogenic injury in the growing population of older people with diabetes. Our study is novel in that it specifically examines the relationship between diabetes and ADRs and the impact of ADRs on outcomes in a multimorbid older population, as well as its large sample size with participants from six centres across Europe. The method of ascertainment of ADRs was substantially more detailed and rigorous compared to that used in most other studies that rely on routinely collected clinical data.

This study has some limitations. We could not reliably distinguish between Type 1 and Type 2 diabetes from the trial dataset, which might have provided additional insights into ADRs in hospitalised older diabetic patients. Additionally, we lacked sufficient data on the duration and control of diabetes, both factors which could influence the degree of ADR risk especially as some patients could simply be maintained on diet control and may not be on any anti-diabetic medication. Finally, incident ADRs may theoretically have been affected by the trial intervention, which sought to minimise ADRs. However, few recommendations from the trial intervention were adopted by clinicians looking after participants and the trial results showed no impact on ADRs [24] so this is unlikely to be significant. Nevertheless, further research addressing the above-mentioned limitations would help confirm and build on our findings.

## Conclusions

In summary, hospitalised multimorbid older people with diabetes are at a significantly higher susceptibility for developing specific ADRs, especially AKI, which increases their risk of mortality. Along with diabetic control for preventing vascular complications including renal damage, efforts to reduce polypharmacy, regular medication review and deprescribing of nephrotoxic medications and more cautious use of diuretics are recommended to reduce the AKI ADR rates and improve outcomes in this growing population.

### Supplementary Information

Below is the link to the electronic supplementary material.

Supplementary file1 (DOCX 59 KB)
