# Supplementary material for: Diabetes mellitus increases risk of adverse drug reactions and death in hospitalised older people: the SENATOR trial
Source: Eur Geriatr Med. 2023 Dec 21;15(1):189–99. doi: 10.1007/s41999-023-00903-w (PMC10876722; doi:10.1007/s41999-023-00903-w)
Supplement: Supplementary file 1 — Supplementary file1 (DOCX 59 KB) [file 41999_2023_903_MOESM1_ESM.docx]

**Supplementary Table 1**: Definitions of pre-specified ADRs, obtained from the SENATOR protocol (22).

| Event | Definition |
| --- | --- |
| Acute bleeding | Melaena or haematuria or haematemesis or haemoptysis with or without a drop in haemoglobin level > 2 g/dl (not due to rehydration) or associated symptoms (hypotension, tachycardia, pallor) or secondary renal failure |
| Acute diarrhoea | New liquid stools reported by the patient, or the nursing staff or new liquid stools detected by medical staff on physical examination or new liquid (non-solid) stools occurring more than 3 times in 24 h |
| New major constipation | Subjective symptoms of hard stools and/or less than 3 bowel movements per week and/or supported by nursing records |
| Acute dyspepsia/nausea/vomiting | Subjective symptoms of acute ‘indigestion’/‘upset stomach’ or acute abdominal pain or acute refusal to eat or acute heartburn/acid reflux or acute nausea/vomiting |
| Acute kidney injury | An increase in serum creatinine by 0.3 mg/dl (26.5 μmol/l) within 48 h or an increase in serum creatinine by 1.5 baseline, which is known or presumed to have occurred within the prior 7 days |
| Symptomatic hypoglycaemia | Symptoms with a blood glucose of < 3.5 mmol/L or < 63 mg/dl |
| Acute delirium | Confirmed by a reliable witness and the DSM-V criteria. Supported by a 4AT ≥ 4 and/or MMSE < 23/30 |
| Fall/s | New fall/s |
| Major serum electrolyte disturbance | A sodium (Na) of < 130 mmol/l or > 145 mmol/l and/or a potassium (K+) < 3.5 mmol/l or > 5.2 mmol/l and/or a corrected calcium (Ca++) < 2.1 mmol/l or > 2.7 mmol/l |
| Symptomatic bradycardia | Heart rate of < 50 beats with symptoms |
| Symptomatic orthostatic hypotension | A systolic blood pressure drop ≥20 mmHg ± diastolic blood pressure drop ≥10 mmHg within 3 min of standing from the lying or sitting posture associated with symptoms |
| New onset unsteady gait | New onset of unsteady gait that results in poor mobility and unsteady balance |
| Unspecified adverse event | For ADRs not specified above e.g., liver failure, anaphylaxis |

1080 unadjudicated prevalent ADRs

1063 ADRs sent for adjudication

17 excluded as missing data on both causality and severity

773 prevalent ADRs

290 excluded as non-eligible ADRs

***Supplementary Figure 1:*** *Flowchart demonstrating the adjudication process for prevalent ADRs.*

1281 unadjudicated incident ADRs

1049 ADRs sent for adjudication

232 excluded as missing data on both causality and severity

828 incident ADRs

221 excluded as non-eligible ADRs

***Figure 2:*** *Flowchart demonstrating the adjudication process for incident ADRs.*

**Supplementary Table 2:** Comparison of causality of prevalent ADRs between people with and without diabetes.

| **Prevalent ADRs** | **Total no. of prevalent ADRs**  **(n = 773)**  **n (%)** | **Prevalent ADRs in people with diabetes**  **(n = 284)**  **n (%)** | | **Prevalent ADRs in people without diabetes**  **(n = 489)**  **n (%)** | | **p-value** | |
| --- | --- | --- | --- | --- | --- | --- | --- |
|  |  | **Indeterminate/Unlikely/ possible** | **Probable/ certain** | **Indeterminate/Unlikely/ possible** | **Probable/ certain** | |  |
| Acute bleeding | 80 (10.3%) | 4 (1.4%) | 16 (5.6%) | 29 (5.9%) | 31 (6.3%) | | 0.05 |
| Acute diarrhoea | 30 (3.9%) | 7 (2.5%) | 7 (2.5%) | 7 (1.4%) | 9 (1.8%) | | 0.18 |
| New onset constipation | 42 (5.4%) | 5 (1.8%) | 6 (2.1%) | 13 (2.7%) | 18 (3.7%) | | 0.22 |
| Acute dyspepsia/nausea/vomiting | 59 (7.6%) | 8 (2.8%) | 9 (3.2%) | 22 (4.5%) | 20 (4.1%) | | 0.30 |
| Acute kidney injury (AKI) | 82 (10.6%) | 24 (8.5%) | 9 (3.2%) | 32 (6.5%) | 17 (3.5%) | | 0.32 |
| Symptomatic hypoglycaemia (SH) | 14 (1.8%) | 1 (0.4%) | 12 (4.2%) | 1 (0.2%) | 0 | | <0.001 |
| New onset fall/S | 110 (14.2%) | 25 (8.8%) | 11 (3.9%) | 56 (11.5%) | 18 (3.7%) | | 0.58 |
| Delirium | 39 (5.0%) | 11 (3.9%) | 3 (1.1%) | 23 (4.7%) | 2 (0.4%) | | 0.92 |
| Major serum electrolyte disturbance | 176 (22.8%) | 39 (13.7%) | 30 (10.6%) | 53 (10.8%) | 54 (11%) | | 0.23 |
| Symptomatic bradycardia | 25 (3.2%) | 2 (0.7%) | 4 (1.4%) | 4 (0.8%) | 15 (3.1%) | | 0.24 |
| Symptomatic orthostatic hypotension | 17 (2.2%) | 4 (1.4%) | 3 (1.1%) | 4 (0.8%) | 6 (1.2%) | | 0.60 |
| New onset unsteady gait | 19 (2.5%) | 6 (2.1%) | 3(1.1%) | 10 (2%) | 0 | | 0.33 |
| Unspecified adverse event | 80 (10.3%) | 22 (7.7%) | 13 (4.6%) | 31 (6.3%) | 14 (2.9%) | | 0.10 |

ADRs: Adverse Drug Reactions

**Supplementary Table 3:** Comparison of causality of incident ADRs between people with and without diabetes.

| **Incident ADRs** | **Total no. of incident ADRs**  **(n = 828)**  **n (%)** | **Incident ADRs in people with diabetes**  **(n = 334)**  **n (%)** | | **Incident ADRs in people without diabetes**  **(n = 494)**  **n (%)** | | **p-value** | |
| --- | --- | --- | --- | --- | --- | --- | --- |
|  |  | **Indeterminate/Unlikely/ possible** | **Probable/ certain** | **Indeterminate/Unlikely/ possible** | **Probable/ certain** | |  |
| Acute bleeding | 68 (8.2%) | 14 (4.2%) | 12 (3.6%) | 19 (3.8%) | 23 (4.7%) | | 0.58 |
| Acute diarrhoea | 65 (7.9%) | 8 (2.4%) | 19 (5.7%) | 13 (2.6%) | 25 (5.1%) | | 0.27 |
| New onset constipation | 138 (16.7%) | 24 (7.2%) | 17 (5.1%) | 31 (6.3%) | 66 (13.4%) | | 0.16 |
| Acute dyspepsia/nausea/vomiting | 68 (8.2%) | 15 (4.5%) | 12 (3.6%) | 21 (4.3%) | 20 (4%) | | 0.42 |
| Acute kidney injury (AKI) | 99 (11.9%) | 20 (6%) | 27 (8.1%) | 26 (5.3%) | 26 (5.3%) | | 0.008 |
| Symptomatic hypoglycaemia (SH) | 14 (1.7%) | 0 | 14 (4.2%) | 0 | 0 | | <0.001 |
| New onset fall/S | 22 (2.7%) | 7 (2.1%) | 3 (0.9%) | 10 (2%) | 2 (0.4%) | | 0.31 |
| Delirium | 44 (5.3%) | 12 (3.6%) | 7 (2.1%) | 19 (3.8%) | 6 (1.2%) | | 0.26 |
| Major serum electrolyte disturbance | 163 (19.7%) | 16 (4.8%) | 39 (11.7%) | 30 (6.1%) | 78 (15.8%) | | 0.69 |
| Symptomatic bradycardia | 11 (1.3%) | 1 (0.3%) | 1 (0.3%) | 4 (0.8%) | 5 (1%) | | 0.24 |
| Symptomatic orthostatic hypotension | 16 (1.9%) | 2 (0.6%) | 4 (1.2%) | 5 (1%) | 5 (1%) | | 0.84 |
| New onset unsteady gait | 3 (0.4%) | 1 (0.3%) | 0 | 1 (0.2%) | 1 (0.2%) | | 1.00 |
| Unspecified adverse event | 117  (14.1%) | 30 (9%) | 29 (8.7%) | 23 (4.7%) | 35 (7.1%) | | 0.006 |

**ADRs:** Adverse Drug Reactions

Presence of diabetes

Mortality

Mortality

Presence of diabetes

Acute kidney injury

**Step 1**

B = 0.41

SE = 0.21

p=0.047

**Step 2**

B = - 0.55

SE = 0.21

p=0.008

**Step 3**

B = 0.36

SE = 0.21

OR = 1.43

p=0.08

**Step 3**

B = - 0.90

SE = 0.30

p=0.003

a

b

c

**Supplementary Figure 3:** Mediation analysis between the presence of diabetes and mortality with acute kidney injury as the mediator. B: unstandardised beta. SE: standard error.

Presence of diabetes

Mortality

Presence of diabetes

Mortality

eGFR

**Step 1**

B = 0.41

SE = 0.21

p=0.047

**Step 2**

B = - 7.48

SE = 1.53

p<0.001

**Step 3**

B = 0.32

SE = 0.21

p=0.13

OR = 1.38

**Step 3**

B = 0.008

SE = 0.004

p=0.028

a

b

c

**Supplementary Figure 4:** Mediation analysis between the presence of diabetes and mortality with eGFR as the mediator. B: unstandardised beta. SE: standard error.

**Supplementary Table 4:** Mediation analysis between having diabetes and mortality with incident acute kidney injury and eGFR as mediators - results of direct and indirect effect and Sobel's test.

| Mediators | Indirect effect | Direct effect | Sobel test |
| --- | --- | --- | --- |
| Acute kidney injury (incident) | 0.50 | 0.36 | 0.048 |
| eGFR | - 0.06 | 0.32 | 0.06 |
